# Effects of Manganese on Genomic Integrity in the Multicellular Model Organism *Caenorhabditis elegans*

**DOI:** 10.3390/ijms222010905

**Published:** 2021-10-09

**Authors:** Merle M. Nicolai, Ann-Kathrin Weishaupt, Jessica Baesler, Vanessa Brinkmann, Anna Wellenberg, Nicola Winkelbeiner, Anna Gremme, Michael Aschner, Gerhard Fritz, Tanja Schwerdtle, Julia Bornhorst

**Affiliations:** 1 Food Chemistry, Faculty of Mathematics and Natural Sciences, University of Wuppertal, Gaußstraße 20, 42119 Wuppertal, Germany; merle.nicolai@uni-wuppertal.de (M.M.N.); ann-kathrin.weishaupt@uni-wuppertal.de (A.-K.W.); anna.reimer@uni-wuppertal.de (A.G.); 2 TraceAge—DFG Research Unit on Interactions of Essential Trace Elements in Healthy and Diseased Elderly (FOR 2558), Berlin-Potsdam-Jena-Wuppertal, 14558 Nuthetal, Germany; baesler@uni-potsdam.de (J.B.); winkelbeiner@uni-potsdam.de (N.W.); tanja.schwerdtle@uni-potsdam.de (T.S.); 3 Department of Food Chemistry, Institute of Nutritional Science, University of Potsdam, Arthur-Scheunert-Allee 114–116, 14558 Nuthetal, Germany; 4 Institute of Toxicology, Medical Faculty, Heinrich Heine University Düsseldorf, Moorenstr.5, 40225 Düsseldorf, Germany; vanessa.brinkmann@uni-duesseldorf.de (V.B.); anna.wellenberg@hhu.de (A.W.); fritz@uni-duesseldorf.de (G.F.); 5 Albert Einstein College of Medicine, Department of Molecular Pharmacology, Neuroscience and Pediatrics, Bronx, NY 10032, USA; michael.aschner@einsteinmed.org; 6 German Federal Institute for Risk Assessment (BfR), Max-Dohrn-Strasse 8–10, 10589 Berlin, Germany

**Keywords:** manganese, oxidative stress, DNA repair, DNA damage response, *Caenorhabditis elegans*

## Abstract

Although manganese (Mn) is an essential trace element, overexposure is associated with Mn-induced toxicity and neurological dysfunction. Even though Mn-induced oxidative stress is discussed extensively, neither the underlying mechanisms of the potential consequences of Mn-induced oxidative stress on DNA damage and DNA repair, nor the possibly resulting toxicity are characterized yet. In this study, we use the model organism *Caenorhabditis elegans* to investigate the mode of action of Mn toxicity, focusing on genomic integrity by means of DNA damage and DNA damage response. Experiments were conducted to analyze Mn bioavailability, lethality, and induction of DNA damage. Different deletion mutant strains were then used to investigate the role of base excision repair (BER) and dePARylation (DNA damage response) proteins in Mn-induced toxicity. The results indicate a dose- and time-dependent uptake of Mn, resulting in increased lethality. Excessive exposure to Mn decreases genomic integrity and activates BER. Altogether, this study characterizes the consequences of Mn exposure on genomic integrity and therefore broadens the molecular understanding of pathways underlying Mn-induced toxicity. Additionally, studying the basal poly(ADP-ribosylation) (PARylation) of worms lacking poly(ADP-ribose) glycohydrolase (PARG) *parg-1* or *parg-2* (two orthologue of PARG), indicates that *parg-1* accounts for most of the glycohydrolase activity in worms.

## 1. Introduction

Exposure to the transition metal and essential trace element manganese (Mn) occurs both naturally and anthropogenically. Natural sources of Mn are diverse and ubiquitous with Mn being the 12th most abundant element in the Earth’s crust. Mn does not usually exist in its elemental form, but is found as silicates, carbonates, and oxides [1]. The element can exist in several oxidative states, with Mn(II) and Mn(III) being the most common forms in biological systems [2]. Due to the rich natural occurrence of Mn in vegetables, cereal products, and drinking water, the adequate intake of 3 mg/day for adults is generally met [3]. Therefore, Mn deficiency has not been observed in humans [4]. Continuous scientific progress and rising industrial processes have led to increased release of Mn into the environment, which causes short- and long-term environmental and health risks [5]. Although the essential trace element is needed as a component for several enzyme systems [6,7], overexposure is associated with various toxicity endpoints in humans [8]. For the general population, chronic overexposure due to contaminated drinking water or food is of the greatest concern. Bioaccumulation of the metal can further increase the Mn concentration in specific regions or food items. An example is the significant bio-concentration of Mn in water by aquatic biota, which are taken up by marine and freshwater plants, phytoplankton, aquatic invertebrates, and finally fish [5]. In addition to natural sources, emission from mining, Mn alloy, production, welding, coke oven, dry battery manufacturing, Mn salt production, gasoline additive (MMT), and Mn-containing agrochemicals increase regional soil and water Mn concentrations to new extreme levels [1,9]. Currently, South Africa, Russia, Gabon, Australia, and Brazil have the richest deposits of Mn [5]. High concentrations of inhaled Mn, mostly in an occupational setting, cause acute toxicity in the respiratory system. Even higher nutritional Mn exposure arises from long-term parenteral nutrition therapy [10,11] or in formula-fed infants [12,13].

Mn overexposure is associated with neurodegeneration and can have a negative impact on movement, cognition, emotion, and behavioral responses [14,15]. Recently, several epidemiological studies have indicated an association between environmental exposure to Mn and neurological effects in children and adults. It has been observed that Mn accumulates in the dopamine-rich region of the basal ganglia, causing dopaminergic neurodegeneration [16,17,18]. In fact, in vivo extrapolations suggest a 1–5-fold increase in the Mn concentration in this region of the human brain under occupational exposure, causing dopaminergic neurodegeneration [19,20]. Animal models have been used extensively to study the pathobiology of neurotoxicity by applying translationally human-relevant Mn concentrations [21]. Additionally, in vitro models have been used to understand Mn uptake, homeostasis, and toxicity to single cell lines. However, effective concentrations are not comparable between different cell lines and are not capable of reflecting the situation in a whole (human) organism. Despite the published investigations, little is known about the underlying mechanisms of neurodegeneration. A proposed mechanism for the pathological changes and symptoms of Mn overexposure is the induction of oxidative stress [22]. Excessive production of reactive oxygen and nitrogen species (RONS), either directly via a Fenton-like reaction or indirectly by inhibiting the respiratory chain in mitochondria, leads to increased interactions with macromolecules such as DNA [23]. Recent evidence from in vitro studies suggests that Mn may damage the DNA or disturb cellular DNA damage response pathways under conditions of either overload due to high exposure or disturbed homeostasis [24]. The resulting DNA damage in combination with an insufficient DNA repair system might contribute to the neurological dysfunction [25,26]. However, the results regarding the genotoxic potential of Mn are inconsistent and further studies are needed to clarify whether Mn species are genotoxic. The soil-dwelling nematode *Caenorhabditis elegans* (*C. elegans*) is a well-established model organism used for investigating developmental biology, aging, neurobiology, and genetic toxicology, as well as toxicity testing. Approximately 60–80% of *C. elegans* genes have human homologs and most DNA repair pathways found in mammals are conserved in the worm [27,28,29,30]. Easy genetic manipulation and the short life cycle allow us to use forward genetics to gain knowledge about specific genes involved in the DNA damage response. Accordingly, this study was conducted to address these open questions in an in vivo model organism to gain further insight into the underlying mechanisms of Mn-induced adverse effects, focusing on genomic integrity by means of DNA damage and DNA damage response.

## 2. Results and Discussion

### 2.1. Excessive Mn Exposure Causes Concentration- and Time-Dependent Increase of Mn Content and Lethality

**Figure 1:**
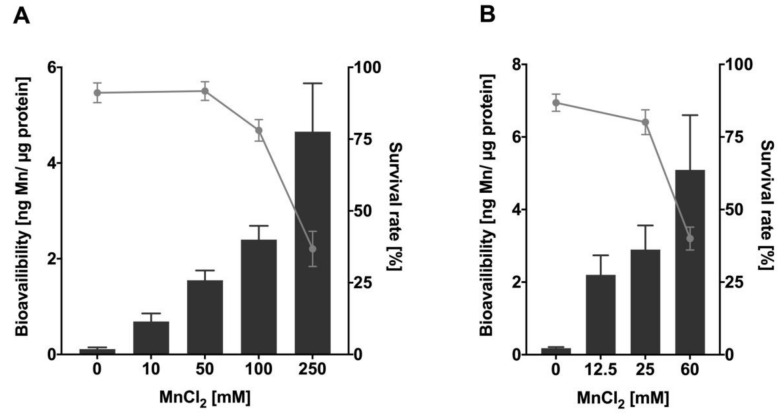
Mn bioavailability and lethality of N2 (WT) after Mn overexposure. Results show dose- and time-depending survival curves (%) and concentration-dependent Mn uptake (ng Mn/µg protein) of L4 stage N2 (WT) following (**A**) 1 h and (**B**) 4 h MnCl_2_ exposure. Survival of worms was quantified 24 h after exposure and bioavailability was measured analytically via ICP-OES. Data are expressed as means ± SEM of at least four independent experiments.

To investigate Mn uptake after acute Mn exposure, L4 *C. elegans* wild-types were incubated with MnCl_2_ both for 1 h and 4 h at various concentrations between 0–250 mM MnCl_2_ or 0–60 mM MnCl_2_, respectively. The results presented in Figure 1 indicate dose- and time-dependent uptake of Mn that inversely correlates with the survival rate of the worms. Nematodes incubated with 250 mM MnCl_2_ for 1 h show a total Mn content of ~4.7 ng Mn/µg protein (Figure 1A). N2 (WT) exposed to MnCl_2_ for 4 h reach similar total body concentrations at 60 mM MnCl_2_ (approximately 1/4th of the corresponding concentration for 1 h) (Figure 1B). Lethality increases at 100 mM MnCl_2_ (1 h) and above, reaching an approximated LD_50_ at 200 mM MnCl_2_ for 1 h. Lethality testing after 4 h exposure indicates an LD_50_ value of ~50 mM MnCl_2_. Similar lethality results were already seen by Neumann et al. [26] where the dosing regime was comparable, whereby small varieties might be caused by different laboratory settings [21]. All further experiments were conducted with nematodes exposed to MnCl_2_ at sub-toxic to toxic concentrations (in detail: up to 250 mM MnCl_2_ for 1 h (survival 37 +/− 6%); up to 60 mM MnCl_2_ for 4 h (survival 40 +/− 4%)), as genotoxic chemicals are expected to induce decreased genomic integrity at doses that do not trigger extensive cell death and might follow a non-linear dose–response relationship in genotoxicity testing [31,32].

### 2.2. Mn Causes a Decrease in Genomic Integrity and the Formation of Oxidative DNA Damage at Sub-Toxic and Toxic Concentrations

**Figure 2:**
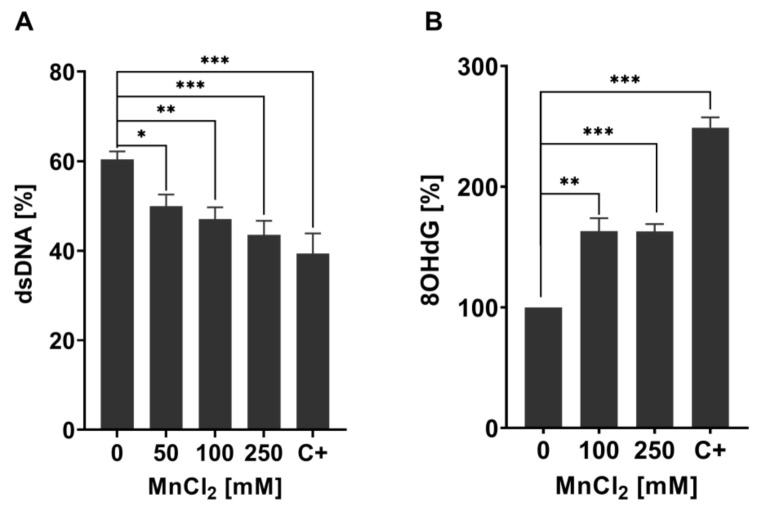
Changes in genomic stability-related markers in Mn exposed N2 (WT) *C. elegans*. (**A**) Percentage of dsDNA to total DNA concentration of N2 (WT) *C. elegans* treated with MnCl_2_ for 1 h. As the positive control, 40 µM Bleomycin for 1 h was used (C+). (**B**) Relative 8OHdG levels of N2 (WT) exposed to 100 mM and 250 mM MnCl_2_ for 1 h. As a positive control, 6.5 mM tBOOH was used (C+). Data are expressed as means ± SEM of at least three independent experiments. For statistical analysis, the unpaired *t*-test was performed. *: *p* < 0.05, **: *p* < 0.01, and ***: *p* < 0.005 compared to respective untreated N2 (WT).

Mn-induced toxicity is implicated to be mediated by induction of oxidative stress [33,34], which can cause increased interactions of RONS with macromolecules such as DNA [35,36]. Oxidative DNA damage is one of the main mutagenic events in germline cells and genomic integrity is especially important as *C. elegans* germline is the site of cell mitosis, meiosis, and oocyte maturation [37,38,39]. Nevertheless, genomic integrity is equally important in post-mitotic cells, which are found outside of the mature nematode’s germline and are irreversibly withdrawn from the cell cycle [40,41]. We decided to investigate the DNA damage in total worms, therefore looking into global effects of Mn on the organism´s genome without differentiating between cell types or tissues, especially since post-mitotic cells incur DNA damage over a protracted time [41]. As our studies show that the bioavailability at LD_50_ values for 1 h and 4 h was proportional to time and concentration (Figure 1), we decided to focus on the investigation of DNA damage after 1 h MnCl_2_ exposure. Previous studies also showed that 1 h Mn exposure already causes adverse outcomes for various oxidative stress endpoints in *C. elegans*, suggesting that this exposure scenario could also lead to DNA damage [26]. Initially, we focused on quantifying DNA strand breaks, which are caused directly by exogenous or endogenous sources or resulting as DNA repair intermediate and can cause loss of information and mutations, DNA-protein crosslinks, and can also have strong effects on the secondary DNA structure [42,43,44]. For quantification, the alkaline unwinding method was utilized, which uses the percentage of dsDNA out of total DNA as a marker for genomic integrity. DNA strand breaks provoke DNA strands to unwind at those sites in an alkaline environment, leading to a decrease of dsDNA. Results of the alkaline unwinding assay show a significant dose-dependent decrease of the percentage of dsDNA after 1 h Mn exposure, starting at sub-toxic concentrations (50 mM MnCl_2_) (Figure 2A). At 250 mM, MnCl_2_ dsDNA is reduced from 60% to 40%, indicating an increase of DNA strand breaks and a decrease of genomic integrity. DNA strand breaks have multiple upstream causes, one source may be oxidative DNA modifications or apurinic/apyrimidinic (AP) sites formed during DNA repair [36,45].

**Figure 3:**
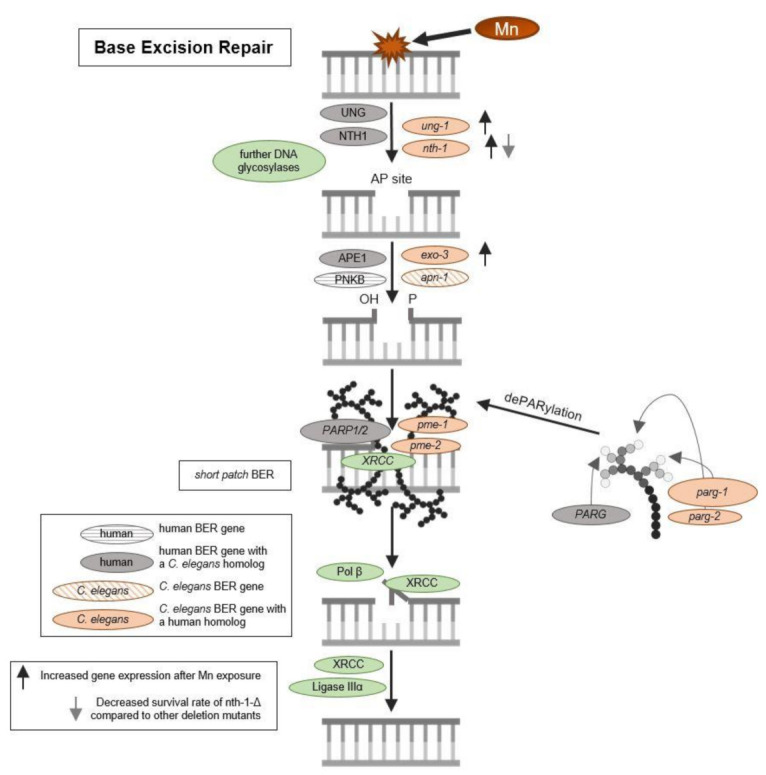
Schematic overview of base excision repair and DNA damage response. BER is initiated by DNA N-glycosylases, which cleave the glycosylic bond linking the altered (oxidized pyrimidic) base and the deoxyribose, resulting in AP site formation. AP-endonucleases will then remove the base-free deoxyribosylphosphate. The resulting single nucleotide gap can be filled either by short patch BER or long patch BER. In the case of short patch BER, the single-nucleotide gap is simply filled and ligated by DNA polymerase β and DNA ligase. If long patch BER occurs, polymerase δ or ε will replace part of the damaged DNA strand beyond the gap by extending the 3´strand. A flap endonuclease will then remove the displaced DNA strand and the remaining nick will be ligated by a DNA ligase III. PARylation facilitates the recruitment of XRCC and other BER enzymes and PAR chains are removed again by PARGs.

Oxidative DNA modifications (and also other base modifications) are repaired via BER (see Figure 3). The damaged base is, in both mammals and worms, excised by glycosylases, leaving AP sites, which are then processed further to DNA single-strand breaks by endonucleases before being repaired by further BER repair enzymes [46,47,48]. To investigate the origin of DNA strand breaks and potential RONS-induced oxidative DNA damage after Mn exposure, 8OHdG was measured utilizing the OxiSelect^TM^ Oxidative DNA Damage ELISA kit. Guanine is likely the most intensely investigated DNA base for oxidative DNA damage, as its low oxidation potential makes it particularly susceptible to incur RONS-induced damage [49,50]. The interaction of singlet oxygen with guanine leads to the formation of 8-oxo-7,8-dihydroguanine (8oxodG) and 8OHdG, which are in equilibrium with each other and are equally used as biomarkers in both in vitro and in vivo studies [51]. The base modification disrupts cellular functions by altering protein–DNA binding and the DNA secondary structure, which can cause changes in genome stability, gene regulation, and telomere protection [52,53,54,55]. Ahn et al. [56] proved that 8OHdG was shown to be a reliable oxidative DNA damage marker in nematodes. Here, silver nanoparticles caused an increase of the base modification, demonstrating that 8OHdG formation also occurs in *C. elegans* under oxidative stress conditions. The method, as applied by Ahn et al. [56], was improved by quantifying the content of dC and a respective isotopically labeled IS (dC-IS) after hydrolysis using HPLC-MS/MS. This allowed normalization to the respective hydrolysis rate as well as the actual DNA content. A significant increase in damage was induced by a 1 h Mn exposure to 100 mM and 250 mM MnCl_2_ (Figure 2B). Differences between those two doses were not detectable. These findings of decreased genomic integrity (increase of DNA strand breaks and oxidatively modified DNA bases) caused by Mn are in line with studies performed in neuroblastoma cells (SH-SY5Y) [57] and further corroborate the results of Yang et al. [58], which show that Mn induces increased levels of 8OHdG in the striatum of mice. Other investigations in mammals and (neuronal) cell lines come to the same conclusion [24,41], but studies performed by Sava et al. and Oikawa et al. suggested that Mn causes an increase of 8oxodG levels of neuronal cells (PK-12), but only if co-incubated with dopamine or melanin [59,60]. Another in vitro study was performed on Mn-toxicity resistant cells (SCOV-3 clones) and showed that these cells have an increased ability of DNA repair, by means of enhanced PARP and AP endonuclease activity compared to the non-resistant original cell line. This indicates that formation of Mn-induced DNA damage is compensated by the higher DNA repair levels of those cells [61].

### 2.3. Increased Mn-caused DNA Damage Does Not Lead to the Formation of Apoptotic Bodies

**Figure 4:**
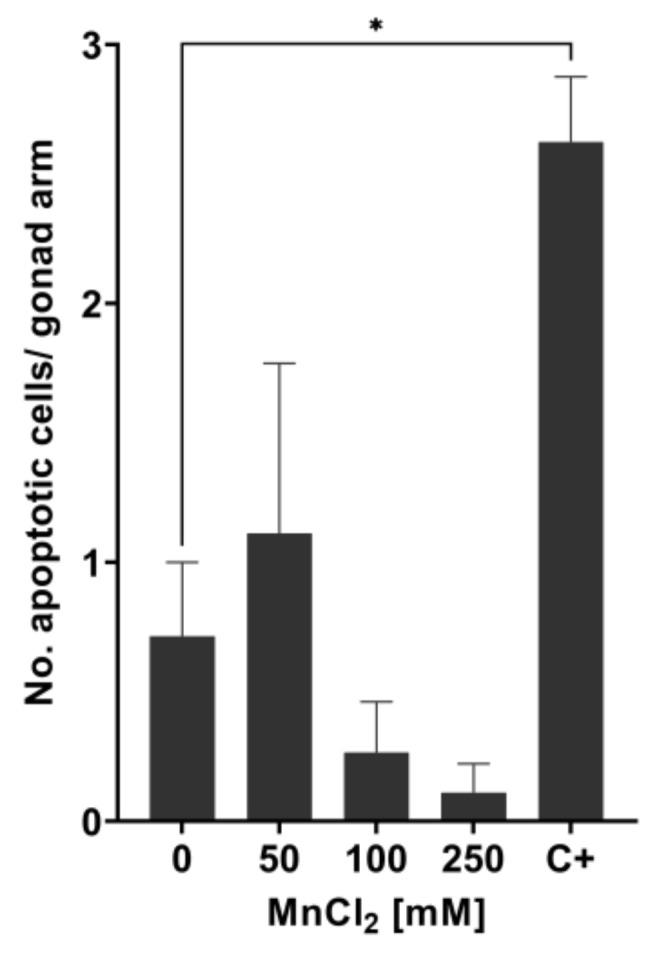
Measurement of apoptotic bodies in N2 (WT) *C. elegans* using acridine orange staining. The number of apoptotic cells per gonad arm was determined 24 h after treating wild-type *C. elegans* with MnCl_2_ at sub-toxic and toxic concentrations. As positive control, 200 J/m^2^ UV-C radiation was used (C+). Data are expressed as means ± SEM of two independent experiments, with ≥ 10 worms per sample. For statistical analysis, the unpaired *t*-test was performed. *: *p* < 0.05 compared to respective untreated N2 (WT).

We then proceeded to examine if those doses of Mn causing increased lethality and genotoxic events also lead to the formation of apoptotic bodies in the N2 (WT). Surprisingly, acridine orange staining showed no induction in the formation of apoptotic bodies with incubations of 50 mM, 100 mM, or 250 mM MnCl_2_ for 1 h (Figure 4). This suggests that other cell death pathways lead to the increased lethality after Mn overexposure. An amount of 200 J/m^2^ UV-C was used as the positive control and induced apoptosis as anticipated.

### 2.4. Gene Expression Studies Indicate Activation of BER after Mn Exposure in Addition to Slight Modification of AP Site Incision Activity

**Figure 5:**
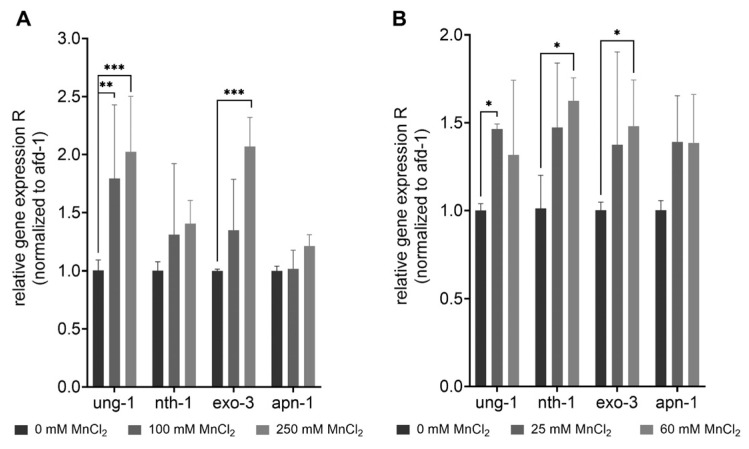
Increase of gene expression of BER-involved genes after excessive MnCl_2_ exposure. N2 (WT) were exposed to MnCl_2_ for (**A**) 1 h (0 mM, 100 mM, and 250 mM) and (**B**) 4 h (0 mM, 25 mM, and 60 mM) and the relative gene expression of *ung-1*, *nth-1*, *exo-3* and *apn-1* compared to non-treated worms was analyzed. Data are expressed as means ± SEM of four independent experiments. For statistical analysis, the two-way ANOVA with Tukey´s multiple comparisons test was performed. *: *p* < 0.05, **: *p* < 0.01, and ***: *p* < 0.005 compared to respective untreated N2 (WT).

DNA damage caused by Mn-induced oxidative stress may further trigger an induction or dysregulation of DNA repair pathways. BER is the main pathway of DNA repair of oxidative DNA damage in mammals [62], and most likely also in *C. elegans* [47]. The repair pathway is highly conserved in the nematode and functional homologs exist for most repair enzymes (see Figure 3). After 1 h exposure, gene expression studies showed a significant increase in the uracil-glycosylase *ung-1* (homolog to human UNG) and AP-endonuclease *exo-3* (homolog to human APE1) at 100 mM and 250 mM MnCl_2_ compared to non-exposed worms (Figure 5). The 4 h incubation with 25 mM and 60 mM MnCl_2_ caused a significant increase in gene expression of *ung-1*, the N-glycosylase *nth-1* (homolog to human NTH1), and *exo-3*, which all act in the initiation of the BER. While in mammals, NTH1 is responsible for the detection and removal of oxidized pyrimidines, it is assumed that the *C. elegans* homolog *nth-1* can also detect and remove oxidized purines, such as 8OHdG. A homolog for the glycosylase OGG1, that removes 8OHdG in mammals, is lacking in *C. elegans* [63,64].

**Figure 6:**
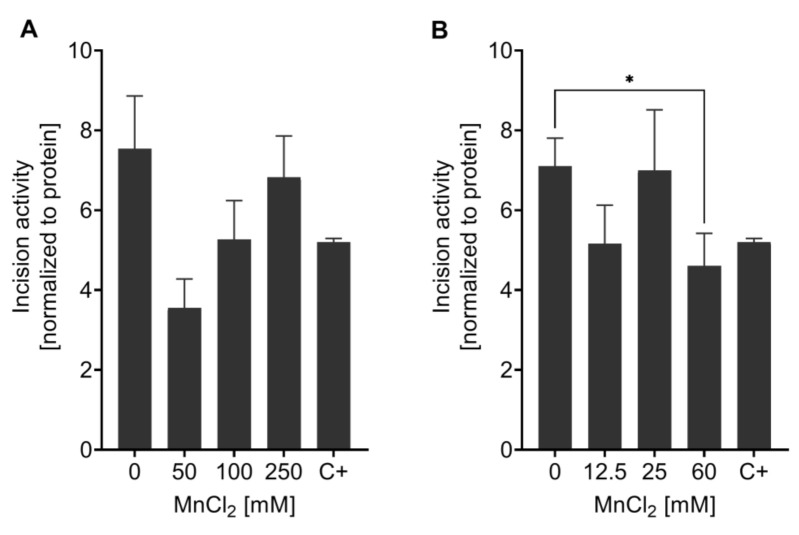
Measurement of AP site incision activity after Mn exposure. BER incision activity was determined towards an AP site analogue containing oligonucleotide by non-radioactive incision activity assay in N2 (WT). Worms were exposed to MnCl_2_ for (**A**) 1 h (0 mM, 50 mM, 100 mM, and 250 mM) and (**B**) 4 h (0 mM, 12.5 mM, 25 mM, and 60 mM). Olaparib was used as a positive control (C+) at 200 µM for 1 h. Data are expressed as means ± SEM of four independent experiments. For statistical analysis, the unpaired *t*-test was performed. *: *p* < 0.05 compared to respective untreated N2 (WT).

The incision activity assay was applied to study the effect of Mn on the capacity to incise AP sites after excessive exposure to the trace element. No significant changes were observed after 1 h Mn exposure with doses of 50 mM, 100 mM, or 250 mM MnCl_2_ (Figure 6A). After the 4 h incubation with Mn, at the highest concentration (60 mM MnCl_2_) incision activity towards the AP site containing oligonucleotide was significantly reduced (63 ± 7.5%) compared to non-exposed N2 (WT) (Figure 6B). Olaparib was used as positive control. In general, not much data regarding DNA repair after Mn exposure is published, especially when looking into incision activity of nematodes. While BER incision activity was measured in mice with varying trace element status [65], the effect of Mn alone on DNA repair efficiency has yet to be studied. Other studies focus on the sensitivity of different BER deficient species/cells towards Mn, but not the adverse consequences of Mn on DNA repair (e.g., [66]). Further research is therefore called for, especially considering the importance of BER in post-mitotic cells such as neurons [41]. A failure of DNA damage in those cells might be associated with neurodegeneration after genotoxic events.

### 2.5. Reverse Genetic Studies Indicate a Slight Phenotype of the nth-1(Δ) Mutant and Significant Differences in parg-1 and parg-2 Activity

**Figure 7:**
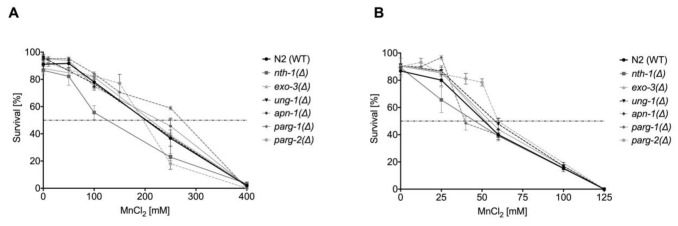
Dose–response curves of MnCl_2_ on BER and DNA damage response-deletion mutants compared to wild-type *C. elegans*. Worms were incubated with various concentrations of MnCl_2_ for (**A**) 1 h or (**B**) 4 h. Data are expressed as means ± SEM of at least three independent experiments.

To investigate the individual roles of BER-involved enzymes in response to Mn, the knock-down strains *ung-1(Δ)*, *nth-1(Δ)*, *exo-3(Δ)*, and *apn-1(Δ)* were examined for distinct Mn-dependent phenotypes. The induction of oxidative stress by Mn exposure observed in wild-type *C. elegans* by Neumann et al. 2019 [26] was also observed in the investigated deletion mutants defective in a single BER protein (Supplementary Materials, Figure S1), but Mn concentration-dependent differences between the strain were not detectable. However, changes in lethality after excessive Mn exposure were observed for the *nth-1(Δ)* strain. A functional knock-down of the N-glycosylase *nth-1* caused higher sensitivity towards Mn following 1 h exposure (Figure 7A). This supports the hypothesis that *nth-1* in *C. elegans* may also be capable of detecting and excising 8OHdG as discussed above. However, no differences in the survival rate were observed after 4 h incubation with MnCl_2_. Further studies found that the *exo-3(Δ)* strain is hypersensitive to other oxidizing agents, reflected in decreased life span and brood size, developmental delay, and abnormal vulval organogenesis [46,67], but lethality data shown here do not capture the sensitivity of that strain under the given conditions.

**Figure 8:**
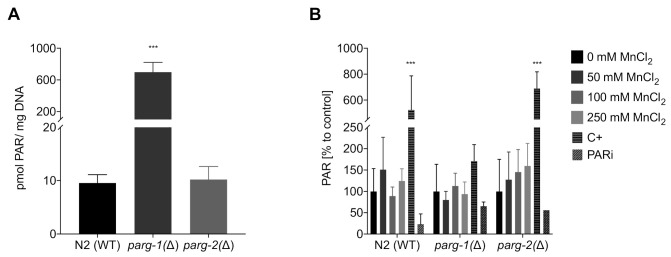
Determination of PARylation levels via HPLC-MS/MS. (**A**) Comparison of the basal PARylation levels in non-treated N2 (WT), *parg-1(Δ)*, and *parg-2(Δ)* worms. (**B**) Relative PARylation levels of N2 (WT), *parg-1(Δ)*, and *parg-2(Δ)* worms treated with 1 h MnCl_2_ at sub-toxic and toxic concentrations. PAR levels are normalized to the DNA amount and compared relatively to the respective untreated control. An amount of 6.5 mM tBOOH (1 h) was used as positive control (C+) and 100 µM Olaparib (1 h) as PAR inhibitor (PARi). Data are expressed as means ± SEM of at least three independent experiments. For statistical analysis, the unpaired *t*-test was performed. ***: *p* < 0.005 compared to respective untreated N2 (WT).

For investigations regarding the DNA damage response, we build on the studies performed by Neumann et al. 2019 [26], who investigated PARylation in N2 (WT) and *pme-1(Δ)* (orthologue of human PARP1) and *pme-2(Δ)* (orthologue of human PARP2) strains. These results show that after 1 h and 4 h of incubation Mn does not exert any effects of PAR induction in wild-type *C. elegans*. While deletion of the genes, responsible for the induction of PARylation, caused an overall basal reduction of R-Ado levels, exposing *pme-1(Δ)* worms to 250 mM MnCl_2_ for 1 h caused a significant increase of PARylation [26]. While the poly(ADP-ribose) polymerases PARP-1 and 2 both have obvious roles in DNA repair by binding to the DNA damage site and post-translationally modifying proteins to form “recruitment platforms” for other proteins needed for DNA repair, such as XRCC1 [68,69], it is also discussed that the poly(ADP-ribose) glycohydrolase PARG co-operates to facilitate downstream cellular DNA repair processes [70]. PARG is responsible for the degradation of PAR, releasing monomeric ADP-ribose moieties [71], which might be needed for DNA damage response at different damage sites. It is also posited, that once PARylation caused the recruitment of XRCC1, removal of the PAR chain facilitates the translocation of XRCC1 from PAR directly to the DNA strand break and increases the repair kinetics of downstream processes [72]. In contrast to humans, worms have two functional orthologues of the glycohydrolase [73,74]. Evaluation of basal PAR levels of these two PARG deletion mutant strains, *parg-1(Δ)* and *parg-2(Δ)*, showed significant differences between the glycohydrolase activities (Figure 8A). A knock-down of *parg-1(Δ)* caused basal PAR levels to increase 70-fold (699 pmol PAR/mg DNA) compared to wild-type and *parg-2(Δ)* (10 pmol PAR/mg DNA). This data suggest that the main degradation activity is exerted by *parg-1* and that *parg-2* is not able to compensate for a deficiency. These results are corroborative of those by Janisiw et al., which showed by immunostaining that *parg-1* has the main PAR glycohydrolase activity in the *C. elegans* germline [75]. Present knowledge on the functional role of *parg* is scarce, especially in the context of global effects in whole organisms. When exposing those three strains (*parg-1(Δ), parg-2(Δ),* and N2 (WT)) to Mn, a significant induction of PAR was not observed (Figure 8B). PAR levels of the wild-type strain N2 (WT) and mutant strain *parg-2(Δ)* were comparable, while PARylation in *parg-1(Δ)* was both less inducible by the positive control *t*BOOH as well as less suppressed by the PAR inhibitor Olaparib. This is most likely due to the already high basal PAR levels in this strain.

## 3. Materials and Methods

### 3.1. *C. elegans* Maintenance and Exposure to Mn

The *C. elegans* wild type N2 (WT) and the deletion mutant strains *nth-1(Δ)* (ok724), *ung-1(Δ)* (tm2862), *parg-1(Δ)* (gk120), and *parg-2(Δ)* (ok980) (all from *Caenorhabditis* Genetics Center (CGC), Minneapolis, MN, USA) and *exo-3(Δ)* (tm4374), *apn-1(Δ) (tm6691)* (Mitani laboratory, National BioResource Project, Tokyo, Japan) were cultivated on *E. coli*-covered 8P plates at 20 °C as described in previous studies and by Brenner 1974 [76,77]. After synchronization, eggs were allowed to hatch overnight and L1 larvae were seeded on NGM plates with OP50 *E. coli* as the food source. Worms were allowed to reach L4 larvae stage without further interference. For Mn exposure, 3000 synchronized L4 worms per sample were incubated in liquid (85 mM NaCl) with MnCl_2_ (99.9% Manganese(II) chloride tetra-hydrate, 203734, Sigma-Aldrich, St. Louis, MO, USA) for 1 h and 4 h in the absence of *E. coli* at different concentrations between 0–250 mM MnCl_2_. Afterwards, worms were washed at least three times with 85 mM NaCl + 0.01% Tween, before shock-freezing the pellets in liquid nitrogen for storage at −80 °C or directly proceeding with the following experiments.

### 3.2. ICP-OES Measurement of Mn Bioavailability

For analysis of Mn uptake, inductively coupled plasma-optical emission spectrometry (ICP-OES (Spectro, Kleve, Germany)) was used. Frozen samples were homogenized by three freeze-thaw cycles and sonication (UP100H ultrasonic processor (Hielscher, Teltow, Germany), 3 × 20 s, 100% amplitude, highest setting), dried, and finally, acid-assisted digested (HNO_3_:H_2_O_2_; 1:1) at 95 °C. The following device parameters were chosen for the analytical measurements. Plasma power: 1400 W, refrigerant gas flow: 12 L/min, auxiliary gas flow: 1 L/min, nebulizer gas type and flow: MicroMist, 1 L/min, and wavelength: 257.611 nm. The purity of the plasma torch argon was greater than 99.99%. For quantification, an external calibration was conducted using a multi-element mix (Spetec-645) and measurement accuracies were assessed using single-cell protein standards certified for trace elements (Commission of the European Communities, Community Bureau of References, BCR). For analysis, the Spectro Smart Analyzer software was used, and all samples were normalized to protein content measured by BCA analysis (bicinchoninic acid assay-kit (Thermo Scientific, Walthalm, MA, USA)).

### 3.3. Lethality Studies after Mn Exposure

The acute toxicity of Mn was determined by lethality testing. After 1 h and 4 h treatment, a known number of worms was transferred to OP50-seeded NGM plates. Alive and dead worms were manually counted 24 h after the treatment. Animals that did not respond to the mechanical stimulus of touch (using a platinum/zirconium wire) were considered as dead.

### 3.4. Measurement of DNA Damage after Mn Exposure Utilizing Alkaline Unwinding

The adapted alkaline unwinding assay was employed as described previously [78]. Briefly, worms were placed in 1 mL alkaline unwinding buffer (AU buffer; 0.5 M NaH_2_PO_4_, 0.5 M Na_2_HPO_4_, 0.1 M EDTA, pH 7.5) after Mn treatment and were made assailable to the alkaline solution by using slight sonication (1 mL AU buffer, 2 × 20 s on lowest setting, 100% amplitude). After centrifugation and removal of the supernatant, 1.5 mL alkaline solution (0.9 M NaCl, 10 mM Na_2_HPO_4_, 0.03 N NaOH in dH_2_O) was added to all samples. Unwinding was conducted at room temperature (RT) in the dark for exactly 15 min, before neutralizing the solution with 0.1 N HCl, sonication on ice (15 s, highest setting), and adding SDS to a final concentration of 0.05%. The single- and double-stranded DNA were separated by successional elution of 0.15 M and 0.35 M potassium phosphate buffer over 1 mL hydroxyapatite columns at 60 °C. The amount of the single-stranded DNA (ssDNA) and double-stranded DNA (dsDNA) fractions was determined using Hoechst stain (Hoechst 33258 nucleic acid stain) at a final concentration of 7.5 × 10^−7^ M and the fluorescence was measured using a microtiter fluorescence reader (Infinite Pro; 360 nm excitation wavelength and 455 nm emission wavelength; Tecan, Männedorf, Switzerland). The percentage of dsDNA indicates the genomic integrity of the worms as in alkaline environments, ssDNA occurs at sites of DNA strand breaks, and higher levels of dsDNA correlate with higher levels of genome integrity. The anti-cancer drug bleomycin was used as positive control at a concentration of 40 µM for 1 h.

### 3.5. ELISA Measurement of 8OHdG

The OxiSelect^TM^ Oxidative DNA Damage ELISA kit (Cell Biolabs, Inc., San Diego, CA, USA) was used for the rapid detection and quantification of 8-hydroxyguanine (8OHdG) in DNA samples obtained from N2 (WT) pellets, as described by Ahn et al. 2014 [56] but with some modifications as described in the following. The DNA was isolated from 3000 animals each, using the Qiagen Tissue and Blood DNA extraction kit, following manufacturer’s instructions. DNA content was measured using a NanoDrop, samples were aliquoted to ~40 µg DNA/sample. After vacuum drying the samples, enzymatic hydrolysis was used to get mononucleotides. For this, dried DNA samples were dissolved in 10 µL dH_2_O and butylated hydroxytoluene (BHT) at a final concentration of 6.5 mM was added to each sample. Then, dsDNA was separated into single DNA strands by incubating all samples for 3 min at 100 °C while shaking. Immediate cooling on ice for another 2 min allows the DNA to re-form double helixes, but more loosely than before. At this point, 2.5 µL of 50 µM desoxy-cytosine (dC) internal standard (IS) was added to each analyte for later normalization. An amount of 5 µL Na-Succinate/CaCl_2_ (100 mmol/ 50mmol/L, pH 6) buffer was added before pipetting 1.6 µL 0.556 U/µL micrococcus nuclease to each sample. The mixture was vortexed thoroughly before adding 2.5 µL 0.001 U/µL phosphodiesterase. All samples were vortexed once again and shortly centrifuged. Enzyme incubation was conducted at 37 °C overnight. The next day, 2 µL 1 U/µL alkaline phosphatase was added and incubated for another 2 h at 37 °C. Lastly, samples were filtered using a 10 kDa cut-off filter (Nanosep 10K, Pall, Port Washington, NY, USA), and samples were centrifuged for 20 min at 13,000 rpm at RT. A total of 2 µL of each sample was used for cytosine quantification via HPLC-MS/MS for normalization (as described by Finke, Winkelbeiner et al. 2020 [79]), the rest was used for the semi-quantitative measurement of 8OHdG via the competitive inverse ELISA kit. Manufacturer’s instructions were followed, and all requisite reagents were included in the kit. As positive control, 6.5 mM *tert*-butyl hydroperoxide (*t*BOOH) was used.

### 3.6. Measurement of Apoptotic Bodies

For determination of apoptosis, early L4 larvae were incubated with MnCl_2_ for 1 h in liquid followed by a 24 h regeneration period on a lawn of *E. coli* covered NGM plates. For measurement of the apoptotic bodies, worms were incubated with acridine orange as described in Gartner et al. [80], and individual nematodes were anesthetized with 15 mM sodium azide as described before. Worms were examined under the fluorescence microscope (Olympus CJX41, Olympus, Tokyo, Japan) at 40× magnification and apoptotic bodies were counted in the posterior germline loop. As the positive control, 200 J/m^2^ UV-C irradiation was used.

### 3.7. Quantitative Real-Time PCR Analysis for Gene Expression Studies

Gene expression studies were performed using TaqMan^TM^ Gene expression assays. RNA was isolated using the TRIzol^®^ method from incubated worm pellets as described previously (33). For cDNA synthesis utilizing the High Capacity cDNA Reverse Transcription Kit (Applied Biosystems), 1 µg of isolated RNA was used. The quantitative real-time PCR (BioRad, Hercules, CA, USA) using TaqMan^TM^ Gene expression Assay probes (Life Technologies, Carlsbad, CA, USA) for each gene was used and *afd-1* (actin homolog) was used as the housekeeping gene for normalization. Fold difference was determined via the comparative 2^−ΔΔCt^ method. The following probes were used: *afd-1* (Assay ID: Ce02414573_m1), *nth-1* (Assay ID: Ce02445360_g1, *ung-1* (Assay ID: Ce02453385_m1), *exo-3* (Assay ID: Ce02423726_g1), and *apn-1* (Assay ID: Ce02435571_g1).

### 3.8. Measurement of Incision Activity for AP Sites

For AP site incision activity analysis, the method of non-radioactive base excision repair (BER) incision activity described by Winkelbeiner et al. 2020 [65] was used. After extract preparation of samples with 3000 worms each and protein quantification via BCA, 5 µg of protein were used for each sample for optimal determination of enzyme activity. Extracts were incubated with the fluorescently labeled DNA lesion containing hairpin-oligonucleotides (AP site analog containing). Using polyacrylamide gel electrophoresis (PAGE), intact and incised oligonucleotides were separated and quantified via fluorescence measurement. The ratio of intact and incised oligonucleotides correlates with the incision activity of glycosylases involved in BER. The PAR polymerase (PARP) inhibitor Olaparib was used as positive control at 200 µM for 1 h.

### 3.9. Analysis of PAR Levels Via HPLC-MS/MS

Preparation of samples for poly(ADP-ribose) (PAR) extraction was conducted with 3000 worms per sample. All steps were conducted as described before [26,81]. In brief, the worm´s cuticles were broken by four freeze-thaw cycles before homogenizing the samples with a tissue disruptor (Qiagen, Hilden, Germany) in 1 mL 20% ice-cold trichloroacetic acid. Samples were washed twice with 70% EtOH and dried at 37 °C. Afterwards, pellets were resuspended in 400 µL 0.5 M KOH and incubated at 37 °C for 50 min. After removal of cell debris, supernatant was neutralized with 4.8 M MOPS (pH 5.9) and DNA concentration was measured via the Hoechst method for later normalization. Then, 2.5 pmol ^13^C,^15^N labeled-PAR was added as an internal standard before digesting the nucleic acids with DNase and RNase, followed by digestion by protein kinase K overnight. Samples were enriched by the High Pure miRNA isolation kit (Roche, Basel, Switzerland) and digested to the monomeric units with alkaline phosphatase and phosphodiesterase I. All enzymes were removed with a 10 kDa cut-off filter (Nanosep 10K, Pall, Port Washington, NY, USA). The analytes were vacuum-dried and resuspended in water for HPLC-MS/MS analysis. PAR analysis was conducted on an Agilent HPLC system (Agilent 1260 Infinity II, Agilent, Santa Clara, CA, USA) coupled to a Sciex triple quadrupole-mass spectrometer (Sciex QTrap 6500+, Sciex, Framingham, MA, USA) equipped with an electrospray ion source operating in positive mode (ESI+). Separation was conducted using a Hypersil Gold aQ 150 mm × 2.1 mm particle size 3 micron and corresponding pre-column and an isocratic flow with 1% ACN + 0.01% formic acid and 99% water + 0.01% formic acid was used. The flow rate was 0.3 mL/min. The following optimized source parameters were used: curtain gas = 35, collision gas = medium, ion spray voltage = 5500, temperature = 600, ion source gas 1 = 40, ion source gas 2 = 40, declustering potential = 66, entrance potential 10, and collision cell exit potential = 8. Quantification was conducted in MRM mode and the transitions of *m/z* = 400 > 136 (collision energy = 27) and *m/z* = 415 > 146 (collision energy = 27) for ribosyladenosine (R-Ado) and ^13^C,^15^N R-Ado, respectively. As a positive control, 6.5 mM *t*BOOH was used (described previously [26]) and 100 mM Olaparib was used as a PARP inhibitor.

### 3.10. Statistical Analysis

Statistical analyses were performed using GraphPad Prism 9 (GraphPad Software, La Jolla, CA, USA). Significance is depicted as *: *p* < 0.05, **: *p* < 0.01, and ***: *p* < 0.005 compared to respective untreated mutant strains or N2 (WT).

## 4. Conclusions

Mn is constantly being introduced into the environment, and rising industrial use of this transition metal causes increased pollution and exposure. It is therefore imperative to understand the short- and long-term environmental and health risks of the exposure to this metal, as well as the underlying pathways of its toxicity. Utilizing the multicellular model organism *C. elegans* allowed us to investigate the adverse effects of Mn. The results of the current study are shedding more light on the underlying mechanisms of Mn-induced toxicity attributing to oxidative stress. Moreover, Mn-induced DNA damage (decreased genomic integrity) interfered with DNA repair and DNA damage responses. The increase in BER gene expression upon Mn exposure implies that the decreased genomic integrity results in activation of BER. PARylation and apoptosis were not triggered by excessive Mn exposure in this study design. To validate the results further, more studies on proteins linked to PARylation are required. Regarding BER specifically, the results show a significant role for *nth-1*, although further investigations are needed to understand the underlying mechanistic pathways. To confirm or refute the hypothesis that observed oxidative DNA damage caused by Mn is associated with the Mn-induced neurodegeneration examined in other studies, neurodegeneration assays are needed.

## Data Availability

Not applicable.

## Supplementary Materials

The following are available online at https://www.mdpi.com/article/10.3390/ijms222010905/s1. Figure S1: Changes of the NAD+/NADH ratio as measure of oxidative stress.
