# Supplementary material for: Effects of Manganese on Genomic Integrity in the Multicellular Model Organism Caenorhabditis elegans
Source: Int J Mol Sci. 2021 Oct 9;22(20):10905. doi: 10.3390/ijms222010905 (PMC8535284; doi:10.3390/ijms222010905)
Supplement: Supplementary file 1 [file ijms-22-10905-s001.zip › ijms-1389342-supplementary.pdf]

Article

Effects of Manganese on Genomic Integrity in the Multicellular Model Organism *Caenorhabditis elegans*

Merle M. Nicolai [1], Ann-Kathrin Weishaupt [1,2], Jessica Baesler [2,3], Vanessa Brinkmann [4], Anna Wellenberg [4], Nicola Winkelbeiner [2,3], Anna Gremme [1], Michael Aschner [5], Gerhard Fritz [4], Tanja Schwerdtle [2,3,6] and Julia Bornhorst [1,2,3,*]

1 Food Chemistry, Faculty of Mathematics and Natural Sciences, University of Wuppertal, Gaußstraße 20, 42119 Wuppertal, Germany; merle.nicolai@uni-wuppertal.de (M.M.N.); ann-kathrin.weishaupt@uni-wuppertal.de (A.-K.W.); anna.reimer@uni-wuppertal.de (A.G.)
2 TraceAge—DFG Research Unit on Interactions of Essential Trace Elements in Healthy and Diseased Elderly (FOR 2558), Berlin-Potsdam-Jena-Wuppertal, 14558 Nuthetal, Germany; baesler@uni-potsdam.de (J.B.); winkelbeiner@uni-potsdam.de (N.W.); tanja.schwerdtle@uni-potsdam.de (T.S.)
3 Department of Food Chemistry, Institute of Nutritional Science, University of Potsdam, Arthur-Scheunert-Allee 114–116, 14558 Nuthetal, Germany
4 Institute of Toxicology, Medical Faculty, Heinrich Heine University Düsseldorf, Moorenstr.5, 40225 Düsseldorf, Germany; vanessa.brinkmann@uni-duesseldorf.de (V.B.); anna.wellenberg@hhu.de (A.W.); fritz@uni-duesseldorf.de (G.F.)
5 Albert Einstein College of Medicine, Department of Molecular Pharmacology, Neuroscience and Pediatrics, Bronx, 10032 NY, USA; michael.aschner@einsteinmed.org
6 German Federal Institute for Risk Assessment (BfR), Max-Dohrn-Strasse 8–10, 10589 Berlin, Germany
* Correspondence: bornhorst@uni-wuppertal.de, Tel.: +49-202-439-3453

Supplementary

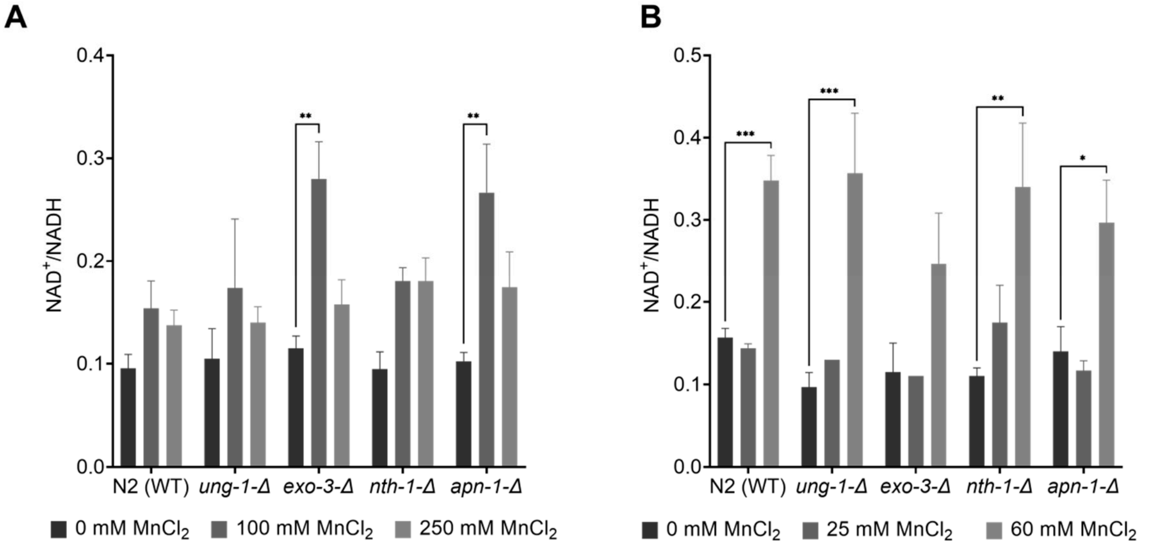

Figure S1. Changes of the NAD+/NADH ratio as measure of oxidative stress. Wild-type, N2 (WT), and BER-deletion mutants (*ung-1-Δ, exo-3-Δ, nth-1-Δ,* and *apn-1-Δ)* were incubated with $MnCl_2$ for (**A**) 1 h or (**B**) 4 h. Data are expressed as means ± SEM of at least three independent experiments. For statistical analysis, the unpaired *t*-test was performed. *: $p < 0.05$, **: $p < 0.01$, and ***: $p < 0.005$.
